# Tobacco smoking and risk of all-cause mortality in Indonesia

**DOI:** 10.1371/journal.pone.0242558

**Published:** 2020-12-01

**Authors:** Holipah Holipah, Hikmawan Wahyu Sulistomo, Asri Maharani

**Affiliations:** 1 Faculty of Medicine Universitas Brawijaya, Malang, Indonesia; 2 Division of Nursing, Midwifery & Social Work, University of Manchester, Manchester, United Kingdom; University of Calfornia San Francisco, UNITED STATES

## Abstract

Tobacco is well known as a risk factor for early morbidity and mortality worldwide. However, the relative risk of mortality and the effects of smoking vary among the countries. Indonesia, as one of the world’s largest market for smoking tobacco, is significantly affected by tobacco-related illness. Previous research has shown that smoking causes several diseases, including stroke, neoplasm and coronary heart disease. There has to date been no research on the hazard risk of smoking for all-cause mortality in Indonesia. This study aimed to identify the association between smoking and all-cause mortality rates in Indonesia. Information from a total of 3,353 respondents aged 40 years and older was collected in this study. The data were taken from the Indonesian Family Life Survey (IFLS) Wave 4 (2007) to collect personal information and determine smoking status and from Wave 5 (2015) to collect information about deaths. Current smokers make up 40.3% of Indonesia’s population. Current smokers were more likely to have a higher risk of all-cause death (hazard ratio = 1.48, 95% confidence interval = 1.11 to 1.98) than non-current smokers. The number of smokers in Indonesia remains high and is expected to increase gradually every year. A firm government policy is needed to reduce the number of smokers in Indonesia which would automatically reduce the health problem of smoking-related illness in the future.

## Introduction

Tobacco smoking is known risk factor for early morbidity and mortality worldwide [1–3]. Smoking is the second largest contributor to global disability-adjusted life-years (DALYs), contributing to 148 million DALYs annually [2]. The World Health Organization (WHO) has estimated that smoking is responsible for 12% and 6% of worldwide deaths among males and females, respectively [4]. It is predicted that one billion people will die in the 21st century as a result of smoking if there is no change in smoking habits, with most of these deaths striking low- and middle-income countries [5].

Prior studies have investigated the association of tobacco and increased mortality rates [6–8], and cohort studies in the UK and US have found that the relative risk of mortality and the effects on the populations vary between countries [9, 10]. Different environmental aspects such as socioeconomic status, stress and genetic factors are among the possible explanations of that variation [11]. The evidence on how smoking increases all-cause mortality rates in low-to middle-income countries is limited. For example, Gu et al. found that 673,000 deaths were attributable to smoking in China in 2005 [12]. In other report, Correa et al. showed that cigarette smoking was responsible for 24,222 (13.64%) deaths of persons aged 35 years and older in 2003 in 16 Brazilian regional capitals [13]. In the Casablanca region of Morocco 9.7% of deaths among people older than 35 were caused by smoking in 2012 [14]. However, less is known regarding the link between smoking prevalence and mortality in Indonesia.

Smoking is responsible for a high proportion of morbidity and mortality in Indonesia. A 2015 study showed that 925,611 males (93.27%) and 66,719 females (6.93%) in Indonesia were hospitalized as a result of diseases attributable to smoking such as hypertension (42.6%), chronic obstructive pulmonary disease (COPD) (40.2%), and stroke other diseases (12%), accounting for 21,05% of all chronic diseases in the country [15]. A 2017 study showed that cerebrovascular attack and ischemic heart disease are leading causes of death in Indonesia. During the period from 2007 to 2017, the mortality rate of diseases attributable to smoking such as a cerebrovascular attack, ischemic heart disease, and COPD increased by 29.2%, 29.0%, and 10.5%, respectively [16].

The incidence of smoking-attributable diseases in Indonesia is predicted to remain high. As of 2016, 39.5% of Indonesian aged 15 years and older are active smokers; this proportion is 7.4% higher than the global average [17]. Furthermore, the percentage of Indonesian men who smoke tobacco has increased dramatically, rising from 56.2% in 2000 to 76.2% in 2015 [17]. As one of the countries that has not signed the World Health Organization Framework Convention on Tobacco Control (WHO FCTC), Indonesia also lacks of policies related to the tobacco market [18, 19]. Taken together, these circumstances indicate that Indonesia has a significant effect on the incidence of tobacco-related illness worldwide.

There has as yet been no research on the hazard risk of smoking for all-cause death in Indonesia. As a developing country with the highest smoking consumption in the world, Indonesia’s patterns are a different from those of other countries, even within Asia [20]. It is essential to determine the relationship between smoking behaviour and the developing risk caused by tobacco smoking. With this study, we aimed to investigate whether smoking status predicts all-cause mortality risk in a developing country.

## Materials and methods

### Study design and sample

The data were derived from the two most recent waves (Wave 4 in 2007 and Wave 5 in 2015) of the Indonesia Family Life Survey (IFLS). The IFLS is a prospective cohort study of the Indonesian population. We have a long-time follow-up because the 8 years gaps between IFLS 4 (2007) and IFLS 5 (2015), and there was no IFLS data collection conducted between those years [21]. The information collected for IFLS includes socio-demographic, lifestyle, and health data as well as blood biomarkers. The survey is conducted by the RAND Corporation (US), the University of Gadjah Mada and SurveyMETER (both in Yogyakarta, Indonesia). Initiated in 1998, the IFLS is representative of 83% of the Indonesian population. The data are freely available from https://www.rand.org/labor/FLS/IFLS.html. All respondents provided written informed consent, and the IFLS was approved by the Institutional Review Boards at RAND (US) and at the University of Gadjah Mada (Indonesia).

This study used information on personal characteristics and smoking status collected in Wave 4 and on deaths in Wave 5 (the first time that mortality information was collected). The household response rate for IFLS Wave 5 was 92%, while the response rate for the individual target households (including split off households as separate) was 90.5%. These re-contact rates are similar to those of most longitudinal surveys in the United States and Europe [21]. As the IFLS is a longitudinal survey, the sampling scheme for the first wave determined the sample in subsequent waves. The first wave sampling scheme stratified 13 of Indonesia’s 26 provinces, then randomly sampled within provinces. A total of 7,730 households were sampled in the first wave. The IFLS implemented a within-household sampling scheme, which included individual interviews with the household head and his/her spouse, up to two of their randomly selected children aged 14 years and younger, a randomly selected individual aged 15 to 49 and his/her spouse, and a randomly selected household member aged 50+ and his/her spouse. The information in both Waves 4 and 5 was provided by the household head and other selected individuals in the household. We included respondents aged 40+ who gave information on smoking status in Wave 4 and for whom survival information was available in the final wave, resulting in 3,353 respondents.

### Research samples, smoking status and outcomes

The samples included in the analysis came from individuals aged 40 years or older at the time of IFLS Wave 4 for whom personal characteristics, smoking status and survival information were available. We included participants aged 40 years and older because health information, including comorbidities, was only available for that age group. The samples were categorized as either current smokers or non-current smokers. A current smoker is defined as a person who was smoking either every day or on some days at the time of the survey. Age at starting to smoke were categorized into ≤ 12, 13–16, and ≥ 17 years old. The outcome measure was all-cause mortality. We used the date of death obtained in Wave 5 to define the duration of survival. The survival time was measured in months from the date of birth. Survival information was derived from month of interview or of death as reported by proxy respondents, typically family members or relatives. We used all-cause mortality because no reliable information on cardiovascular events and cardiovascular mortality is available; this, in turn, results from the fact that there is no connection between the IFLS and hospital records.

A total of 509 deaths occurred in the eight-year period between Waves 4 and 5, with 274 among the 1,475 men (18.5%) and 235 among the 1,878 women (12.5%).

### Covariates

We used the information at baseline for the covariates. We entered age as age group and sex as an indicator (0 as male and 1 as female). We classified the levels of education completed by respondents as less than college or college or higher. Marital status was classified into single as the reference, married and separated/widowed. We used expenditure per capita to measure wealth and assigned respondents to quintiles with the poorest quintile as the reference. We determined the presence of comorbidities based on subjective reports of doctor`s diagnoses and treatment history (answers to the questions ‘Are you now taking the following treatments for […] and its complications?’). The comorbidities included in this study were cardiovascular diseases (CVD), diabetes, and stroke. Respondents were defined as having hypertension if they had a history of hypertension diagnosed by a physician, were on hypertension treatment, or had a systolic blood pressure ≥140 mmHg or a diastolic blood pressure ≥90 mmHg. Blood was processed to obtain biomarker levels. Respondents were considered to have low high-density lipoprotein (HDL) cholesterol if they had an HDL cholesterol measurement of ≤ 35 mg/dL and to have high total cholesterol if they had a total cholesterol measurement of ≥200 mg/dL. We included central obesity as covariate: waist circumference >90 cm in men and >80 cm in women.

### Statistical analyses

Categorical baseline characteristics were summarised as frequencies and percentages, with comparisons between death and non-death using chi-square tests. Continuous variables were summarised using means and standard deviations and tested for differences using Kruskal–Wallis tests. The associations between smoking status and all-cause mortality risk among Indonesians were examined using a Cox proportional hazard model with year as the time scale. In the Model 1, we included all participants and used all of the covariates (demographic, socio-economic, behavioural and biomarker covariates) without smoking status. We then included smoking status in Model 2. In the Models 3 and 4, we applied the Cox proportional hazard models separately among current smokers and non-current smokers. We included age at starting to smoke in Model 3. Survival time was entered as a number of years, counted from the birth date to the date of death for deceased respondents and to the date of subsequent interview (IFLS Wave 5) for survivors. IFLS provided survey weight with corrections for attrition to adjust for non-response bias. We used that sampling weight for all descriptive models as well as the Cox proportional hazard models to adjust for non-response and to ensure population representativeness using svyset command in STATA 16. We tested the Cox Proportional Hazards model for central assumption of proportional hazards using Kaplan-Meier Curves (S1 Fig). It shows that the graph of the survival function versus the survival time by smoking status results in a graph with parallel curves, meaning that smoking status as the main predictor is proportional. All analyses were performed using Stata version 16 (College Station, TX). We used the xtreg package in STATA 16 for the survival analysis [22].

## Results

Table 1 presents the statistics on demographic and socio-economic characteristics along with health behaviours by mortality status. The sample consisted of 53.8% females. The respondents’ average age was 56.9 years (standard deviation = 10.4). Only approximately 6% of respondents had completed college. Most of the respondents (78.7%) were married and lived on the islands of Sumatra or Java (90.8%). The respondents’ average waist circumference was 80 cm for males and 82 cm for females. The proportions of respondents with cardiovascular diseases, diabetes and history of stroke were 1.9%, 2.4%, and 0.8%, respectively. More than half of the respondents had low HDL cholesterol or hypertension, and almost 40% had high cholesterol levels.

**Table 1:** Baseline characteristics of study participants.

	Total^a^	Death^a^	Non death^a^	P value^a^	Mortality rate (per 1,000 population)
	(n = 3,353)	(n = 509)	(n = 2,844)		
*Smoking status*, *frequency (%)*				<0.001	
Non-current smoker	1,998 (59.61)	226 (11.3)	1,772 (88.6)		24.15
Current smoker	1,354 (40.39)	253 (18.7)	1,101 (81.3)		15.72
Age at starting to smoke (among current smoker)				0.064	
≤ 12	97 (7.69)	18 (18.72)	79 (81.28)		21.88
13–16	327 (25.83)	76 (23.26)	251 (76.74)		23.73
≥ 17	841.65 (66.48)	135 (16.08)	706 (83.92)		17.80
Age, mean (SD)	56.9 (10.4)	66.0 (10.9)	55.2 (9.4)	<0.001	
*Gender*, *frequency (%)*				<0.001	
Male	1,547 (46.1)	268 (17.3)	1,278 (82.6)		23.22
Female	1,806 (53.8)	211 (11.7)	1,594 (88.2)		15.64
*Area of living*, *frequency (%)*				0.484	
Rural	1,910 (56.9)	266 (13.9)	1,643 (86.0)		18.42
Urban	1,443 (43.0)	213 (14.8)	1,226 (85.2)		19.51
*Graduated from college or higher degree*, *frequency (%)*				<0.001	
Yes	188 (5.6)	9 (4.9)	179 (95.0)		7.02
No	3,164 (94.3)	470 (14.8)	2,694 (85.1)		19.72
*Marital status*, *frequency (%)*				<0.001	
Single	49 (1.4)	16 (7.8)	41 (84.0)		16.67
Married	2,641 (78.7)	327 (12.3)	2,314 (87.6)		16.61
Separated/widowed	663 (19.7)	145 (21.8)	518 (78.1)		28.02
*Wealth*, *frequency (%)*				0.084	
1^st^ quintile (poorest)	802 (23.9)	118 (14.8)	682 (85.1)		20.43
2^nd^	693 (20.6)	109 (15.7)	584 (84.2)		20.86
3^rd^	721 (21.5)	105 (14.5)	616 (85.4)		19.00
4^th^	597 (17.8)	87.3 (14.6)	510 (85.3)		19.67
5^th^ quintile (wealthiest)	540 (16.1)	59.9 (11.0)	480 (88.9)		14.52
*Islands*, *frequency (%)*				0.012	
Sumatera and Java	3,046 (90.8)	429 (14.1)	2,616 (85.8)		18.50
Sulawesi	93 (2.7)	13 (13.8)	80 (86.1)		16.87
East islands	90 (2.6)	16 (18.1)	73 (81.8)		25.35
Kalimantan	69 (2.0)	15 (22.1)	54 (77.9)		27.90
Others	55 (1.6)	5 (9.7)	49 (90.2)		9.06
The presence of comorbidities					
CVD, *frequency (%)*				<0.001	
Yes	64 (1.9)	21 (34.0)	42 (65.9)		44.64
No	3,289 (98.0)	458 (13.9)	2,831 (86.0)		18.43
Diabetes, *frequency (%)*				<0.001	
Yes	82 (2.4)	24 (29.5)	58 (70.4)		35.92
No	3,270 (97.5)	455 (13.9)	2,815 (86.0)		18.52
Stroke, *frequency (%)*				<0.001	
Yes	30 (0.8)	16 (54.2)	14 (45.7)		66.41
No	3,322 (99.0)	463 (13.9)	2,859 (86.0)		18.52
HDL ≤ 35 mg/dL				0.926	
Yes	1,745 (52.8)	251 (14.3)	1,494 (85.6)		18.95
No	1,555 (47.1)	222 (14.3)	1,332 (85.7)		19.21
Cholesterol ≥ 200 mg/dL				0.336	
Yes	1,303 (39.1)	173 (13.2)	1,130 (86.7)		18.04
No	2,023 (60.8)	303 (14.9)	1,720 (85.0)		19.57
Hypertension				<0.001	
Yes	1,675 (51.1)	341 (19.7)	1,386 (80.7)		26.02
No	1,727 (51.5)	139 (8.5)	1,487 (91.4)		11.21
Central obesity				0.010	
Yes	1,216 (36.3)	155 (12.7)	1,060 (87.2)		16.43
No	2,126 (63.6)	322 (15.1)	1,803 (84.8)		20.52

**Note:** ^**a**^ = the analyses were performed using survey weight.

Approximately 40.3% of the respondents were current smokers. Approximately 66% respondents started smoking when they were 17 years or older. The summary of descriptive statistics of the sample by mortality, the bivariate analysis and the mortality rates appear on the right pane of Table 1. The data show that the mortality rates were higher among older people, current smokers, those with less educational attainment, and those with no comorbidities. S1 Table provides the baseline characteristic by smoking status.

During the eight years of follow-up, there were 509 mortalities. Current smokers had a higher risk of all-cause mortality. Of the non-current smokers (n = 2,059), 250 died (12.1%); of the current smokers (n = 1,294), 259 died (20.0%). Table 2 shows the estimated hazard ratio and 95% confidence intervals for mortality in two models. We included demographic, marital status, socio-economic status, area of living, and the presence of comorbidities in the Model 1. We than added smoking status in Model 2. The results in Model 1 shows that being younger and college-educated are related to lower hazard risks of all-cause mortality. Respondents who were married or separated/widowed have lower odds of all-cause mortality than those who were single. Living on Kalimantan Islands, and having hypertension or cardiovascular disease were associated with a higher risk of mortality among all participants. All those associations remained significant when we included smoking behaviour in Model 2. It shows that when demographics and other potential covariates were equal, current smokers had a 48% higher risk of death (95% confidence interval = 1.11 to 1.98) than non-current smokers. We further performed those analyses separately by smoking behaviour in S2 Table; S3 Table, and S1 Fig.

**Table 2:** Hazard ratios of all-cause mortality stratified by smoking status.

	All participants (n = 3,277)			
	Model 1		Model 2	
	HR (95% CI)	P value	HR (95% CI)	P value
Current smokers	-	-	1.48 (1.11 to 1.98)	0.007
*Age*, *reference*: *40–49 years old*				
50–59	1.85 (1.21 to 2.85)	0.005	1.84 (1.20 to 2.82)	0.005
60–69	4.15 (2.71 to 6.37)	<0.001	4.10 (2.68 to 6.28)	<0.001
70–79	9.55 (6.05 to 15.10)	<0.001	9.21 (5.82 to 14.58)	<0.001
≥ 80	14.02 (8.39 to 23.42)	<0.001	13.45 (8.03 to 22.51)	<0.001
Female	0.61 (0.48 to 0.78)	<0.001	0.79 (0.57 to 1.08)	0.143
College or higher degree	0.40 (0.19 to 0.82)	0.012	0.41 (0.20 to 0.83)	0.014
*Marital status*, ref: Single				
Married	0.33 (0.17 to 0.62)	0.001	0.33 (0.18 to 0.61)	<0.001
Separated/widowed	0.40 (0.20 to 0.77)	0.007	0.39 (0.20 to 0.76)	0.006
*Wealth*, *reference*: *1*^*st*^ *quintile (poorest)*				
2^nd^	0.98 (0.73 to 1.31)	0.920	0.99 (0.74 to 1.32)	0.952
3^rd^	0.94 (0.70 to 1.26)	0.686	0.92 (0.69 to 1.24)	0.627
4^th^	0.91 (0.66 to 1.25)	0.575	0.90 (0.65 to 1.23)	0.524
5^th^ quintile (richest)	0.74 (0.52 to 1.06)	0.103	0.74 (0.52 to 1.06)	0.102
Living in urban area	1.19 (0.96 to 1.47)	0.105	1.24 (1.00 to 1.55)	0.045
*Islands*,				
ref: Sumatera and Java				
Sulawesi	0.94 (0.58 to 1.53)	0.817	0.93 (0.57 to 1.50)	0.772
East islands	1.14 (0.82 to 1.58)	0.432	1.18 (0.84 to 1.65)	0.318
Kalimantan	1.56 (1.02 to 2.38)	0.040	1.57 (1.03 to 2.38)	0.032
Others	0.47 (0.16 to 1.40)	0.180	0.45 (0.15 to 1.33)	0.152
*The presence of comorbidities*				
CVD	2.68 (1.55 to 4.64)	<0.001	2.78 (1.61 to 4.79)	<0.001
Diabetes	1.63 (0.98 to 2.72)	0.057	1.67 (1.00 to 2.79)	0.049
Stroke	2.32 (1.24 to 4.32)	0.008	2.60 (1.38 to 4.89)	0.003
Hypertension	1.68 (1.31 to 2.13)	<0.001	1.71 (1.34 to 2.18)	<0.001
HDL ≤ 35 mg/dL	0.90 (0.72 to 1.12)	0.373	0.89 (0.71 to 1.10)	0.295
Cholesterol ≥ 200 mg/dL	0.89 (0.71 to 1.11)	0.317	0.89 (0.72 to 1.11)	0.325
Central obesity	1.05 (0.82 to 1.34)	0.666	1.09 (0.85 to 1.39)	0.491

**Note:** All analyses were performed using survey weight. *Abbreviations*: HR, hazard ratio; CI, confidence interval; BP, blood pressure; CVD, cardiovascular disease; HDL, high-density lipoprotein.

## Discussion

Indonesia has the fourth largest population in the world. 2015 study found that more than 25% of the entire Indonesian population were current smokers [1]. In this study, we found that 40.3% of our respondents were current smokers, 89% of them are men. This finding is higher than the global average: 25% for men and 5.4% for women in the world [1]. After China and India, Indonesia has the largest number of smokers in the world. The number of smokers in these three countries accounts for more than 50% of smokers worldwide. The number of smokers in Indonesia did not decrease over the 15 years from 1990 to 2015. One contributing factor may be of the Indonesian government’s lack of tobacco control policies, including policies on smoking in open spaces, marketing cigarette products, and selling cigarette products. Indonesia is not signatory of or party to the WHO FCTC. The FCTC comprehensively prohibits cigarette promotion, advertising and sponsorship, while the Indonesian government has not banned cigarette advertising. Cigarette companies have a large market in Indonesia, and they can aggressively promote their products. In addition, the price of cigarettes in Indonesia is relatively low compared with other countries; the cigarette tax in Indonesia is far below the WHO recommendation of 70% of retail price. Indonesia's average 2017 excise tax rate was 49.1% of retail price. The Indonesian government has been unable to meet the WHO recommendation as its excise law sets the maximum tobacco excise tax at 57% of retail price. This renders Indonesia one of the world’s largest markets for global cigarette marketing. Smoking-related health problems are increasing gradually along with the number of smokers.

The hazard risk of current smokers in this research is shown to be higher than that of non-smokers. The impact of smoking on increased all-cause mortality rates has been widely acknowledged in previous research [7, 8, 23–25]. Similar to this finding, current smokers have a higher risk of all-cause mortality than non-smokers. A meta-analysis on smokers in other Asian countries shows that Asian male smokers in China, Japan, Korea, Singapore, Taiwan and India have a higher risk of all-cause mortality. That risk factor is increased if the person starts smoking at a young age [26]. The death rate of current smokers in Australia is three times higher than among non-smokers. This finding matches research results in Western countries such as the US and UK. The relative risk (RR) caused by smoking consistently is the 2.8 to 3 over 50 years [27, 28].

Age show significant association with the risk of all-cause mortality. Older people have a higher risk of all-cause mortality than younger people. It is well known that ageing affects mortality because of the accumulation of damage and deterioration at the cell, tissue, organ, and organism levels, ultimately leading to death [29].

Our result indicates that education inversely associated with all-cause mortality. Various previous study showed similar result [30, 31]. The people with high education tend to have a better job and higher income, which make people with high education to set aside money to address health concerns [32]. Moreover, education also influences health awareness and health believes [32].

Marital status also influences the hazard risk of all-cause mortality. Physiological distress plays an important role in explaining this finding. Single people are less likely to have a high survival rate than separated, widowed and married people. Married people tend to have more social advantages. Furthermore, married people are more likely to have support from their partner and family, which leads to healthier life [33].

Our result indicated that people who live in urban area more likely to have higher hazard all-cause mortality rate than people who live in rural area. Urban area has a higher population density than in rural area [34]. High population density related to urban stress such as vandalism, noise from neighborhood, air pollution and low quality [34]. The previous report in Denmark and Japan showed that population density influence mortality rate [35, 36].

People living in Kalimantan Island have a higher risk of all-cause mortality rate compared to residents of other islands. Premature mortality such as stroke, ischemic heart disease, diabetes and COPD, was more frequent in all provinces in Kalimantan than in Indonesia overall [16]. One plausible explanation for this is that Kalimantan has a number of industrial areas which may affect air quality [37, 38].

The presence of comorbidities such as CVD, diabetes, stroke, and hypertension, is more likely to increase all-cause mortality risk. According to the data from the Institute for Health Metrics and Evaluation, CVD, diabetes, stroke and hypertension are included in the top ten diseases causing the death in Indonesia [39]. Individuals with non-communicable diseases are usually asymptomatic and progressive as they are chronic conditions. Poor screening system for non-communicable diseases in Indonesia causes the person to not recognize that they have this disease. Those diseases are often diagnosed if the person already has a symptom when the disease already disturbs the physiology of body and causing a problem in the organs [40]. Non communicable diseases are strongly related with poor health behaviors, such as smoking consumption, diet and drinking alcohol. Changing the behaviors of a patient with non-communicable diseases requires a large effort. This circumstance causes the patient with non-communicable disease to struggle to control their disease, leading to a higher mortality risk [41].

Among smokers, people with lower educational levels, and those who are unmarried are more likely to have a higher risk of mortality; among non-smokers, we observed no such association. Education has an inverse effect on smoking. People with lower educational levels are more likely to be smokers than people with higher educational levels; this result is supported by much previous research [33, 42, 43]. The effect on smoking may be explained by awareness of health facts, awareness of preventive treatment and concern about health, and seeking treatment, all of which are more prevalent in people with more formal education; this group also has greater ease accessing health facilities as those with more education are generally also wealthier. In contrast, people with lower levels of education are less likely to seek assistance at a smoking cessation facility. In families with lower educational levels, several smokers usually live together [44]. Furthermore, people who graduate from college are 17% more likely to try to stop smoking than those with less education [42].

Marital status also influences the hazard risk of all-cause mortality in the smoking group. The spouse is twice as important as a friend or even a sibling in an individual’s decision to quit smoking [45]. Among Hispanic and non-Hispanic-white singles in the US, previous research has shown that those who were single/never married had the highest prevalence of smoking. The same study found that single people experience more psychological distress. Unmarried people suffer more from loneliness and dissatisfaction than married people, and they may try smoking to relieve their stress [46].

Our result shows that individuals with comorbidities, such as CVD, stroke, and diabetes, have higher all-cause mortality risks than those without the comorbidities in the non-smoking group, while in the smoking group there is no significant difference in the mortality risk. The lack of association between chronic diseases and mortality risk among current smokers may due to several plausible explanations. Firstly, the respondents may follow the advice from the doctors and stop smoking when they were diagnosed by chronic diseases. A study in Indonesia showed that current smokers had 26% lower odds of having hypertension, while those who quit smoking had higher odds of having hypertension [47]. The second plausible explanation is the presence of other diseases causing mortality among current smokers, such as lung cancer and chronic obstructive pulmonary disease [48]. Further research to establish this finding is required.

This study has a number of limitations. Firstly, it investigates all-cause mortality risk and its association with smoking behaviour as the data source (IFLS Wave 5) provides no acceptable information on specific causes of mortality, such as lung cancer or cardiovascular events. Another limitation is that the IFLS sample is representative of about 83% of the Indonesian population mostly in the west and central parts of the country. IFLS is not representative of all Indonesian provinces. The survey mostly in the west and central parts of the country, in the wave 5 still and excluded most eastern Indonesian provinces, which are considered underdeveloped compared to their western counterparts. Additional data sets on population health that encompass all Indonesian provinces are available (e.g. Indonesian Socioeconomic Survey [SUSENAS] and Riset Kesehatan Dasar [RISKESDAS]), but these data are not longitudinal and do not provide information on the deaths of their respondents. Despite its limitations, this study has several strengths, including its prospective cohort, represent more than 80% of Indonesian population and the high rate of follow-up.

## Conclusions

In conclusion, our results show that the number of current smokers in Indonesia remains high and that smoking increases the risk of all-cause mortality. This situation is expected to directly impact many health problems in the future. It is therefore essential to review the Indonesia government’s tobacco control policies with regard to many aspects such as tobacco regulation, higher taxes on cigarettes, and regulation of smoking areas. The government must also consider potential interventions in order to encourage smoking cessation among current smokers.

## Supporting information

S1 Fig All-cause mortality, stratified by smoking status. (TIF)

S1 Table Baseline characteristics of study participants by smoking status. (DOCX)

S2 Table Hazard ratios of all-cause mortality stratified by smoking status. (DOCX)

S3 Table Hazard ratios of all-cause mortality among smokers (n = 1,266). (DOCX)

## Abbreviations

DALYs: disability-adjusted life years
WHO: World Health Organization
COPD: chronic obstructive pulmonary diseases
WHO FCTC: WHO Framework Convention on Tobacco Control
IFLS: Indonesian Family Life Survey
CVD: Cardiovascular Disease
HDL: high density lipoprotein
RR: relative risk
SUSENAS: Indonesian Socioeconomic Survey
RISKESDAS: Riset Kesehatan Dasar
