# Supplementary figures and images for: Tobacco smoking and risk of all-cause mortality in Indonesia
Source: PLoS One. 2020 Dec 1;15(12):e0242558. doi: 10.1371/journal.pone.0242558 (PMC7707492; doi:10.1371/journal.pone.0242558)

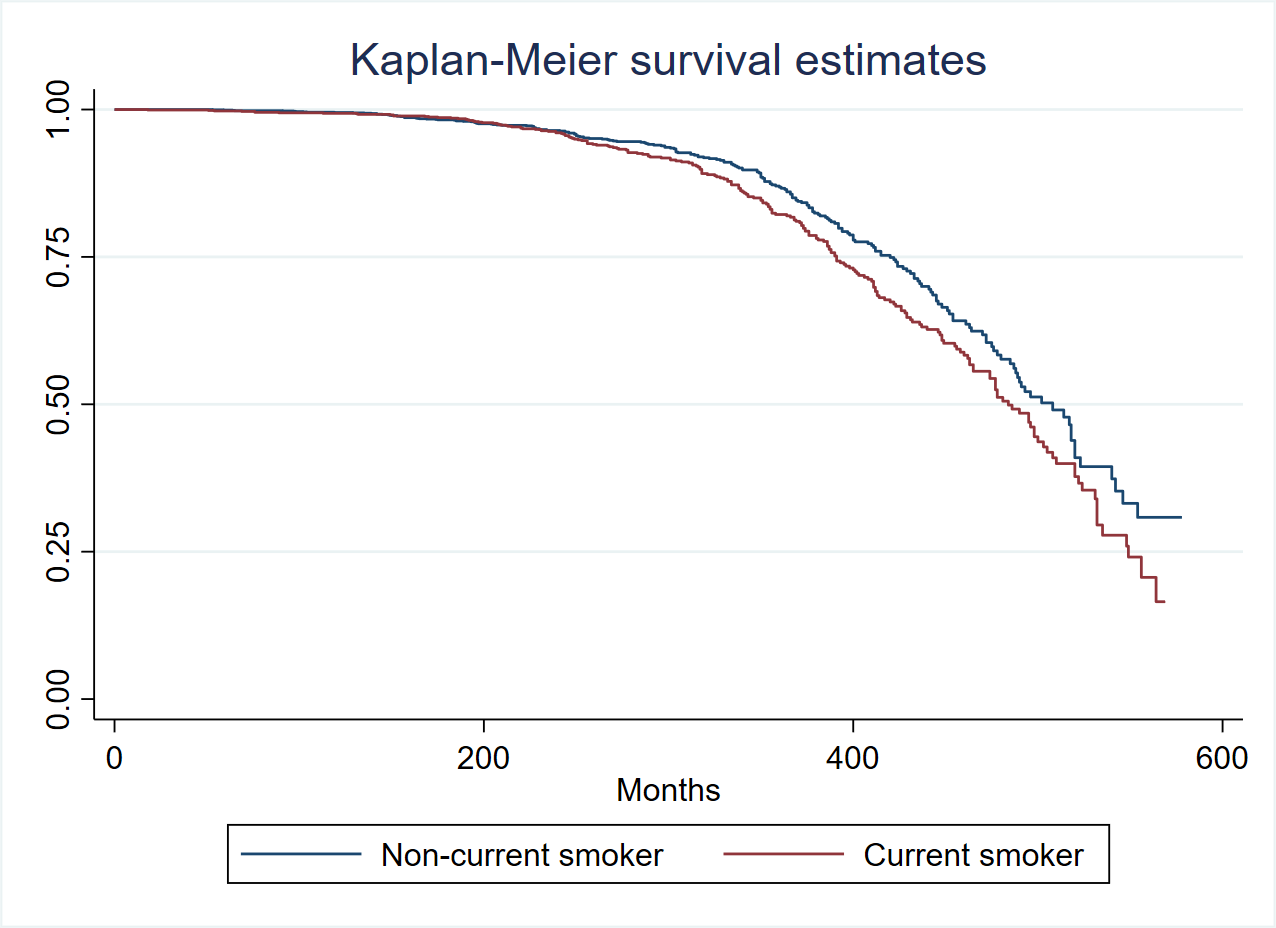

Supplement: S1 Fig — (TIF) [file pone.0242558.s001.tif]
