# Supplementary material for: Tobacco smoking and risk of all-cause mortality in Indonesia
Source: PLoS One. 2020 Dec 1;15(12):e0242558. doi: 10.1371/journal.pone.0242558 (PMC7707492; doi:10.1371/journal.pone.0242558)
Supplement: S1 Table — (DOCX) [file pone.0242558.s002.docx]

**S1 Table.** Baseline characteristics of study participants by smoking status

|  | Total  (n=3,353) | Current smokers  (n=1,354) | Non-current smokers  (n=1,999) | P value |
| --- | --- | --- | --- | --- |
| Age, mean (SD) | 56.9 (10.4) | 55.8 (10.0) | 58.5 (10.8) | <0.001 |
| Female, frequency (%) | 1,806 (53.8) | 150 (11.0) | 1,656 (82.8) | <0.001 |
| Living in an urban area | 1,443 (43.0) | 511 (37.7) | 932 (46.6) | <0.001 |
| College or higher degree | 188 (5.6) | 68 (5.0) | 120 (6.0) | 0.191 |
| *Marital status* |  |  |  | <0.001 |
| Single | 49 (1.4) | 19 (1.5) | 30 (1.5) |  |
| Married | 2,641 (78.7) | 1,173 (86.6) | 1,468 (73.4) |  |
| Separated/widowed | 663 (19.7) | 162 (11.9) | 501 (25.0) |  |
| *Wealth* |  |  |  | 0.162 |
| 1^st^ quintile (poorest) | 802 (23.9) | 339 (25.0) | 463 (23.1) |  |
| 2^nd^ | 693 (20.6) | 281 (20.7) | 412 (20.6) |  |
| 3^rd^ | 721 (21.5) | 301 (22.2) | 420 (21.0) |  |
| 4^th^ | 597 (17.8) | 237 (17.5) | 360 (18.0) |  |
| 5^th^ quintile (wealthiest) | 540 (16.1) | 196 (14.4) | 344 (17.2) |  |
| *Islands* |  |  |  | 0.374 |
| Sumatera and Java | 3,046 (90.8) | 1,242 (91.6) | 1.804 (90.2) |  |
| Sulawesi | 93 (2.7) | 32 (2.3) | 61 (3.0) |  |
| East islands | 90 (2.6) | 30 (2.2) | 60 (3.0) |  |
| Kalimantan | 69 (2.0) | 29 (2.1) | 40 (2.0) |  |
| Others | 55 (1.6) | 22 (1.6) | 33 (1.6) |  |
| CVD | 64 (1.9) | 21 (1.5) | 43 (2.1) | 0.319 |
| Diabetes | 82 (2.4) | 29 (2.1) | 53 (2.6) | 0.213 |
| Stroke | 30 (0.8) | 7 (0.5) | 23 (1.1) | 0.051 |
| HDL ≤ 35 mg/dL | 1,745 (52.8) | 876 (65.5) | 869 (44.2) | <0.001 |
| Cholesterol ≥ 200 mg/dL | 1,303 (39.1) | 415 (30.8) | 888 (44.8) | <0.001 |
| Hypertension | 1,675 (51.1) | 595 (45.4) | 1,080 (54.9) | <0.001 |
| Central obesity | 1,216 (36.3) | 216 (16.0) | 1,000 (50.1) | <0.001 |
