# Supplementary material for: Tobacco smoking and risk of all-cause mortality in Indonesia
Source: PLoS One. 2020 Dec 1;15(12):e0242558. doi: 10.1371/journal.pone.0242558 (PMC7707492; doi:10.1371/journal.pone.0242558)
Supplement: S2 Table — (DOCX) [file pone.0242558.s003.docx]

**S2 Table.** Hazard ratios of all-cause mortality stratified by smoking status

|  | **Smokers (n=1,266)** | | **Non-smokers (n=2,010)** | |
| --- | --- | --- | --- | --- |
|  | **HR (95% CI)** | **P value** | **HR (95% CI)** | **P value** |
| *Age, reference: 40-49 years old* |  |  |  |  |
| 50-59 | 2.52 (1.21 to 5.23) | 0.013 | 1.56 (0.90 to 2.70) | 0.107 |
| 60-69 | 5.48 (2.64 to 11.37) | <0.001 | 3.53 (2.02 to 6.15) | <0.001 |
| 70-79 | 10.31 (4.83 to 22.00) | <0.001 | 8.96 (4.68 to 17.16) | <0.001 |
| ≥ 80 | 17.27 (7.59 to 39.27) | <0.001 | 13.32 (6.44 to 27.55) | <0.001 |
| Female | 0.99 (0.65 to 1.51) | 0.976 | 0.66 (0.43 to 0.99) | 0.050 |
| College or higher degree | 0.15 (0.02 to 0.80) | 0.027 | 0.66 (0.27 to 1.58) | 0.357 |
| *Marital status*, ref: Single |  |  |  |  |
| Married | 0.18 (0.08 to 0.41) | <0.001 | 0.57 (0.24 to 1.87) | 0.451 |
| Separated/widowed | 0.21 (0.08 to 0.52) | 0.001 | 0.75 (0.25 to 2.20) | 0.606 |
| *Wealth, reference: 1^st^ quintile (poorest)* |  |  |  |  |
| 2^nd^ | 1.04 (0.67 to 1.59) | 0.855 | 0.96 (0.64 to 1.45) | 0.878 |
| 3^rd^ | 0.94 (0.63 to 1.42) | 0.801 | 0.86 (0.54 to 1.36) | 0.532 |
| 4^th^ | 0.97 (0.62 to 1.51) | 0.904 | 0.84 (0.52 to 1.35) | 0.485 |
| 5^th^ quintile (richest) | 0.81 (0.50 to 1.32) | 0.414 | 0.69 (0.41 to 1.17) | 0.175 |
| Living in urban area | 1.50 (1.11 to 2.03) | 0.008 | 1.03 (0.75 to 1.41) | 0.831 |
| *Islands*,  ref: Sumatera and Java |  |  |  |  |
| Sulawesi | 1.03 (0.56 to 1.89) | 0.912 | 0.83 (0.38 to 1.84) | 0.663 |
| East islands | 0.97 (0.57 to 1.65) | 0.937 | 1.34 (0.87 to 2.08) | 0.179 |
| Kalimantan | 2.14 (1.31 to 3.49) | 0.002 | 0.93 (0.41 to 2.12) | 0.878 |
| Others | 0.52 (0.13 to 2.02) | 0.352 | 0.31 (0.04 to 2.25) | 0.248 |
| *The presence of comorbidities* |  |  |  |  |
| CVD | 1.47 (0.52 to 4.17) | 0.464 | 3.29 (1.61 to 6.72) | 0.001 |
| Diabetes | 1.31 (0.49 to 3.53) | 0.582 | 1.90 (1.04 to 3.48) | 0.035 |
| Stroke | 1.57 (0.45 to 5.42) | 0.475 | 2.69 (1.21 to 5.95) | 0.014 |
| Hypertension | 1.75 (1.26 to 2.43) | 0.001 | 1.60 (1.10 to 2.33) | 0.013 |
| HDL ≤ 35 mg/dL | 0.74 (0.54 to 1.01) | 0.059 | 1.03 (0.76 to 1.40) | 0.821 |
| Cholesterol ≥ 200 mg/dL | 0.92 (0.65 to 1.28) | 0.627 | 0.89 (0.66 to 1.19) | 0.441 |
| Central obesity | 1.09 (0.71 to 1.65) | 0.682 | 1.17 (0.85 to 1.60) | 0.323 |
