# Supplementary material for: Tobacco smoking and risk of all-cause mortality in Indonesia
Source: PLoS One. 2020 Dec 1;15(12):e0242558. doi: 10.1371/journal.pone.0242558 (PMC7707492; doi:10.1371/journal.pone.0242558)
Supplement: S3 Table — (DOCX) [file pone.0242558.s004.docx]

**S3 Table.** Hazard ratios of all-cause mortality among smokers (n=1,266)

|  |  | |
| --- | --- | --- |
|  | **HR (95% CI)** | **P value** |
| Age at starting to smoke, reference: ≤ 12 |  |  |
| 13-16 | 1.24 (0.73 to 2.11) | 0.409 |
| ≥ 17 | 0.76 (0.46 to 1.25) | 0.288 |
| *Age, reference: 40-49 years old* |  |  |
| 50-59 | 2.49 (1.20 to 5.16) | 0.014 |
| 60-69 | 5.26 (2.54 to 10.89) | <0.001 |
| 70-79 | 9.00 (4.22 to 19.19) | <0.001 |
| ≥ 80 | 21.94 (9.53 to 50.51) | <0.001 |
| Female | 1.15 (0.73 to 1.80) | 0.533 |
| College or higher degree | 0.13 (0.02 to 0.63) | 0.011 |
| *Marital status*, ref: Single |  |  |
| Married | 0.21 (0.10 to 0.47) | <0.001 |
| Separated/widowed | 0.24 (0.10 to 0.58) | 0.002 |
| *Wealth, reference: 1^st^ quintile (poorest)* |  |  |
| 2^nd^ | 1.08 (0.69 to 1.69) | 0.713 |
| 3^rd^ | 0.96 (0.63 to 1.46) | 0.871 |
| 4^th^ | 0.99 (0.62 to 1.59) | 0.999 |
| 5^th^ quintile (richest) | 0.92 (0.56 to 1.51) | 0.750 |
| Living in urban area | 1.56 (1.14 to 2.14) | 0.005 |
| *Islands*,  ref: Sumatera and Java |  |  |
| Sulawesi | 1.18 (0.64 to 2.16) | 0.588 |
| East islands | 0.93 (0.54 to 1.59) | 0.813 |
| Kalimantan | 2.23 (1.35 to 3.68) | 0.002 |
| Others | 0.53 (0.13 to 2.08) | 0.371 |
| *The presence of comorbidities* |  |  |
| CVD | 1.44 (0.52 to 3.99) | 0.475 |
| Diabetes | 1.42 (0.55 to 3.66) | 0.463 |
| Stroke | 3.50 (0.71 to 17.09) | 0.475 |
| Hypertension | 1.46 (0.97 to 2.20) | 0.063 |
| HDL ≤ 35 mg/dL | 0.73 (0.53 to 1.00) | 0.059 |
| Cholesterol ≥ 200 mg/dL | 0.91 (0.65 to 1.30) | 0.637 |
| Central obesity | 0.99 (0.64 to 1.53) | 0.996 |

**Note:** All analyses were performed using survey weight. *Abbreviations:* HR, hazard ratio; CI, confidence interval; BP, blood pressure; CVD, cardiovascular disease; HDL, high-density lipoprotein.
